# Supplementary material for: Antibacterial potential and chromatographic profiling of bioactive compounds from endophytic Streptomyces sp. strain MIRK71 isolated from Mirabilis jalapa (L.)
Source: PeerJ. 2025 Jul 11;13:e19683. doi: 10.7717/peerj.19683 (PMC12258159; doi:10.7717/peerj.19683)
Supplement: Supplemental Information 1 — The first column indicate the MDR pathogens used in the study; the first row represent the different strains of endophyes isolated from Mirabilis jalapa. [file peerj-13-19683-s001.doc]

Supplementary Table1: Primary antimicrobial screening of isolates against 33 clinical MDR pathogens. Diameter of the inhibition zone (in mm)

| Pathogens | MIRK54 | MIRK55 | MIRK56 | MIRK57 | MIRK58 | MIRK59 | MIRK60 | MIRK61 | MIRK62 | MIRK63 | MIRK64 | MIRK65 | MIRK66 | MIRK67 | MIRK68 | MIRK69 | MIRK70 | MIRK71 |
| --- | --- | --- | --- | --- | --- | --- | --- | --- | --- | --- | --- | --- | --- | --- | --- | --- | --- | --- |
| *Escherichia coli* | 16 | - | - | - | - | - | - | - | - | - | - | - | - | - | 5 | - | - | 5 |
| *Escherichia coli* | - | - | - | 2 | 3 | - | - | - | - | - | - | - | - | - | - | - | - | - |
| *Staplylococcus aureus* | 10 | - | - | - | - | - | - | - | - | - | - | - | - | - | 6 | - | - | 8 |
| *Staplylococcus aureus* | - | - | - | - | - | - | - | - | - | - | - | - | - | - | - | - | - | - |
| *Enterococcus faecalis* | 10 | 5 | - | - | - | - | - | - | - | - | - | - | - | - | 9 | - | - | 5 |
| *Staplylococcus aureus* | 4 | - | 4 | - | - | - | - | - | - | - | - | - | - | - | 6 | 16 | - | 8 |
| *Staplylococcus aureus* | - | - | - | - | - | - | - | - | - | - | 7 | - | - | - | - | 10 | - | 18 |
| *Enterococcus faecalis* | - | 7 | - | - | - | - | - | - | - | - | - | - | - | - | - | - | - | 10 |
| *Staplylococcus aureus* | - | - | - | - | - | - | - | - | - | - | - | - | - | - | 5 | - | - | 18 |
| *Staplylococcus aureus* | - | 8 | - | - | - | - | - | - | - | - | - | - | - | - | - | 6 | - | 15 |
| *Enterococcus faecalis* | - | - | 7 | - | - | - | - | - | - | - | - | - | - | - | - | 10 | - | 12 |
| *Staplylococcus aureus* | - | 8 | - | - | - | - | - | - | - | - | - | - | - | - | - | - | - | 12 |
| *Staplylococcus aureus* | - | - | - | - | - | - | - | 3 | - | - | 6 | - | - | - | - | - | - | 6 |
| *Enterococcus faecalis* | - | 5 | - | - | - | - | - | - | - | - | - | - | - | - | 9 | - | - | 8 |
| *Staplylococcus aureus* | - | - | - | - | - | - | - | - | - | - | - | - | - | 2 | - | - | - | - |
| *Klebsiella pneumoniae* | - | 9 | 12 | - | - | - | - | 10 | 16 | - | - | 3 | - | - | 12 | 8 | - | - |
| *Enterococcus faecalis* | - | - | - | - | - | - | - | - | - | - | - | - | - | 6 | 8 | - | - | 11 |
| *Staplylococcus aureus* | - | 7 | - | - | - | - | - | - | - | - | - | 4 | - | - | 9 | - | - | 7 |
| *Enterococcus faecalis* | - | - | - | - | - | - | - | - | - | - | - | - | - | - | - | - | - | - |
| *Staphylococcus aureus* | - | - | - | - | - | - | - | - | - | - | - | 4 | - | 7 | - | - | - | - |
| *Enterococcus faecalis* | - | - | - | 3 | - | - | - | - | - | - | - | - | - | - | - | - | - | - |
| *Staplylococcus aureus* | - | 8 | - | - | - | - | 5 | - | - | - | - | - | - | - | 8 | - | - | 5 |
| *Staplylococcus aureus* | - | 8 | - | - | - | - | 3 | - | - | - | - | - | - | - | - | - | - | 10 |
| *Staplylococcus aureus* | - | - | - | - | - | - | - | - | - | - | - | - | 7 | 7 | - | - | - | - |
| *Staplylococcus aureus* | - | - | - | - | - | - | - | - | - | - | - | - | - | - | 8 | - | - | 6 |
| *Staplylococcus aureus* | - | - | - | - | - | - | - | - | - | - | - | - | 3 | - | 6 | - | - | 5 |
| *Staplylococcus aureus* | - | - | - | - | - | - | - | - | - | - | - | - | - | - | - | - | - | - |
| *Staphylococcus aureus* | - | - | - | - | - | 4 | - | - | - | - | - | - | - | - | - | - | - | - |
| *Staplylococcus aureus* | - | - | - | - | - | - | 3 | - | - | - | - | - | - | - | - | - | - | - |
| *Escherichia coli* | - | 6 | - | - | 10 | - | - | - | - | - | - | 3 | - | - | 8 | - | - | 8 |
| *Staplylococcus aureus* | - | 7 | - | - | - | - | - | - | - | - | - | - | - | - | - | - | - | 9 |
| *Pseudomonas aeruginosa* | - | - | - | - | - | 8 | - | - | - | - | - | - | - | - | - | 9 | - | - |
| *Staplylococcus aureus* | - | - | - | - | - | - | - | - | - | - | - | - | - | - | - | - | - | 10 |

Supplementary Table2: Secondary antimicrobial screening of four isolates against 19 clinical MDR pathogens (in mm)

| **Pathogens** | **MIRK55** | **MIRK68** | **MIRK69** | **MIRK71** |
| --- | --- | --- | --- | --- |
| *Escherichia coli* |  | 3 |  |  |
| *Staplylococcus aureus* |  | 7 |  | 8 |
| *Enterococcus faecalis* | 3 | 3 |  | 4 |
| *Staplylococcus aureus* |  | 9 | 8 | 4 |
| *Staplylococcus aureus* |  |  | 8 | 3 |
| *Enterococcus faecalis* | 3 |  |  | 10 |
| *Staplylococcus aureus* |  | 4 |  | 4 |
| *Staplylococcus aureus* | 6 |  | 5 | 3 |
| *Enterococcus faecalis* |  |  | 8 | 6 |
| *Staplylococcus aureus* | 5 |  |  | 9 |
| *Staplylococcus aureus* |  |  |  | 8 |
| *Enterococcus faecalis* | 4 | 2 |  |  |
| *Klebsiella pneumoniae* | 3 | 2 | 4 |  |
| *Enterococcus faecalis* |  | 5 |  | 8 |
| *Staplylococcus aureus* | 4 | 5 |  | 6 |
| *Staplylococcus aureus* | 5 | 6 |  | 9 |
| *Staplylococcus aureus* | 7 |  |  | 9 |
| *Staplylococcus aureus* |  | 4 |  | 3 |
| *Staplylococcus aureus* |  | 5 |  | 3 |
| *Escherichia coli* | 4 | 4 |  |  |
| *Staplylococcus aureus* | 2 |  |  | 4 |
| *Pseudomonas aeruginosa* |  |  | 2 |  |
| *Staplylococcus aureus* |  |  |  | 2 |
